# NaCTR: Natural product-derived compound-based drug discovery pipeline from traditional oriental medicine by search space reduction

**DOI:** 10.1016/j.csbj.2024.10.035

**Published:** 2024-10-29

**Authors:** Seunghwan Jung, Kwansoo Kim, Seunghyun Wang, Manyoung Han, Doheon Lee

**Affiliations:** Department of Bio and Brain Engineering, KAIST, Daejeon 34141, Republic of Korea

**Keywords:** In silico drug discovery, Natural product, Toxicity, PK, Parkinson's disease

## Abstract

The drug discovery pipelines require enormous time and cost, albeit their infamously high risk of failures. Reducing such risk has therefore been the utmost goal in the process. Recently, natural products (NPs) in traditional oriental medicine (TOM) have come into the spotlight for their efficacy and safety supported throughout the history. Not only that, with the ever-increasing repository of various biological datasets, many data-driven *in silico* approaches have also been extensively studied for better efficient search and testing. However, TOM-based datasets lack information on recently prevalent diseases, while experimental datasets are prone to provide target spaces that are too large. Adequate combination of both approaches can therefore fill in each other's blanks. In this study, we introduce NaCTR, an *in silico* discovery pipeline that achieves such integration to suggest NPs-derived drug candidates for a given disease. First, phenotypes and disease genes for the disease are identified in literature and public databases. Secondly, a pool of potentially therapeutic NPs are identified based on both TOM-based phenotype records and compound-gene interaction datasets. Lastly, the compounds contained in the NPs are further screened for toxicity and pharmacokinetic properties. We use the Parkinson's disease as the case study to test the NaCTR pipeline. Through the pipeline, we propose glutathione and four other compounds as novel drug candidates. We further highlight the finding with literature support. As the first to effectively combine data from ancient and recent repositories, the NaCTR pipeline can be a novel pipeline that can be applied successfully to any other diseases.

## Highlights

• Natural products (NPs) have demonstrated efficacy and safety.
• *in silico* drug discovery pipeline reduces the time and cost for drug development.
• NaCTR is an integrated pipeline of NPs and *in silico* drug discovery pipeline.
• NaCTR effectively reduces search space to find therapeutic compounds.
• NaCTR proposed promising NP treatments for Parkinson's disease.

## Graphical abstract

**Figure:**
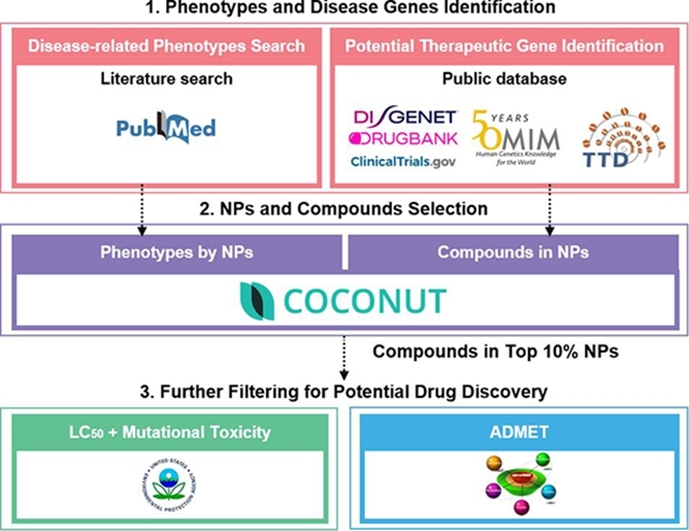

## 1 Introduction

The drug discovery process is a daunting task. Despite the cost of 1 billion US dollars and six years to complete a pipeline of clinical trials to test a single drug [1], only about 10% are successfully approved by the Food and Drug Administration (FDA) [2]. The low success rate usually results from the failures in efficacy [3], possibly due to the lack of genetic evidence between therapeutic targets and diseases [3], [4]. There could also be a problem with the unexpected toxicity or side effects [3], or even with the physicochemical characteristics upon the drug administration, such as pharmacokinetics or pharmacodynamics [3], [5]. The promise of newly developed drugs is further let down by the fact that the drug discovery process has been in the cold “winter” since early 2000s [6], such that there is a significantly smaller number of new drugs and new targets investigated.

In response to these problems, along with the recent advances in the large-scale omics data, there is a growing interest in the search for potential drugs from natural products (NPs) [7], [8]. The usage of these NPs for therapeutic purposes are rooted deeply in human history, especially in the form of traditional oriental medicines (TOMs) from various cultures [9], [10]. An obvious advantage of NPs is that their beneficial effects and harmlessness have been verified when prepared as intended [11], [12], [13]. NP-derived compounds can also offer unique structural characteristics that modern medicine cannot provide. Not only are NP-derived compounds optimized by evolution for specific biological functions [14], they are also known to have higher rigidity that ensures robust binding to their intended targets [15]. It has furthermore been reported that they are more likely to have similar structures to active biological metabolites [16]. Hence, it comes as no surprise that nearly 60% of the small molecule drugs developed since 1981 are NPs or NP-derived synthetic drugs [17], a famous example of which is artemisinin for the treatment of malaria [18].

One of the major problems with modern utilization of TOMs arises when trying to apply them to recently prevalent diseases such as the Parkinson's disease [19], [20]. If a disease was not recorded or even present in the past, then we cannot search for the solution to the term that did not exist. Not only does this limitation severely hinders the applications of traditional knowledge to modern diseases, it also discourages active discovery of compounds with yet unknown therapeutic properties. In order to overcome these cases, it has been recently suggested to convert the modern diseases into a list of symptoms that match the terminologies in TOMs [21], such as ‘tremors’ or ‘muscle rigidity’ for Parkinson's disease. Once the translation is done successfully, further analyses can be investigated for therapeutic possibilities [21], [22].

Meanwhile, in efforts to further reduce the time and cost in experimentally searching for drug candidates, *in silico* drug discovery has come to the fore [23]. This approach is heavily dependent on the datasets available. As such, numerous renowned databases to support each step in drug discovery process were developed. For example, for therapeutic gene identification, disease-related databases like DisGeNET [24], OMIM [25] and TTD [26] provide collective information on disease-related genes. Likewise, for drug candidate compound selection, compound-gene relational databases such as CTD [27] and ChEMBL [28], along with NP-compound databases like FooDB [29] and COCONUT [30] are publicly available. Integration of these databases allows aggressive expansion of the search space for potential compounds in drug discovery.

While such increase in the pool can be helpful, the most of the compound-gene relational data are without actual therapeutic implications. This is to no surprise, considering that many of the drugs proven useful in *in vitro* studies still fail due to the lack of efficacy in the clinical trials [3]. It is therefore up to the researchers to efficiently mine the most relevant compounds.

**Fig. 1:**
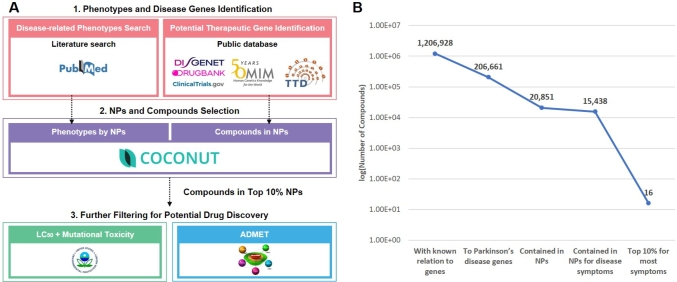
The overall NaCTR pipeline and its effects (A) The overall pipeline to find natural product(NP)-based compounds for a given disease. (B) The line graph illustrating the reduction in the drug search space achieved with the consideration by the NPs. Abbreviation: *LC*_50_, Lethal concentration 50%; ADMET, absorption, distribution, metabolism, excretion, and toxicity model.

Therefore, we aimed to utilize potential therapeutic compounds in TOMs to effectively reduce the size of the compound-gene search space in the drug discovery process. The phenotypic evidences from long history of TOMs could allow safer and more efficient investigation towards more promising set of potential drug candidates. Our Natural product-derived Compound-based drug discovery pipeline from Traditional oriental medicine by search space Reduction (NaCTR) pipeline consists of four steps: target and phenotype identification, TOM-based NP selection for search space reduction, toxicity prediction and pharmacokinetic analyses (Fig. 1). In order to test the validity of the model, the Parkinson's disease was selected as the case study. We show that, upon the identification of therapeutic target genes and disease-related phenotypes, the application of evidences in TOMs reduced the compound-gene search space to the compounds in seven NPs. From further screening by toxicity and pharmacokinetics resulted in five candidates, whose mechanism of action analysis illustrated potential treatment against the Parkinson's disease.

## 2 Materials and methods

### 2.1 Identification of disease-related phenotypes

In order to acquire a list of phenotypes and symptoms related to Parkinson's disease and thereby match them to the terms applicable in Traditional Oriental Medicine (TOM), a literature survey was conducted. Based on a detailed review of the disease, we were able to select 19 phenotypes [31], which consisted of Parkinson's disease and its symptoms.

Them by using herb-phenotype relational datasets available in Compound Combination-Oriented Natural Products Database with Unified Terminology (COCONUT) database [30], only the phenotypes that were associated with the natural products (NPs) in TOM were filtered; i.e. only the terms that have been mentioned and treated in TOM context were acquired. The resulting 17 phenotypes were used for the further analyses. In alphabetic order, the list included anxiety, anxiety disorders, aphasia, bradykinesia, constipation, deglutition Disorders, depressive disorder, dyskinetic syndrome, fatigue, forgetful, mild cognitive disorder, muscle rigidity, pain, postural instability, REM sleep behavior disorder, sense of smell impaired, tremor.

### 2.2 Identification of genes with therapeutic potential

We selected potential therapeutic genes of Parkinson's disease by parsing various databases. The first pool of genes were acquired from disease-gene databases, OMIM [25], TTD [26] and DisGeNET [24]. These databases were carefully selected to take into account various perspectives by which a given gene can be related to a disease of interest.

**Fig. 2:**
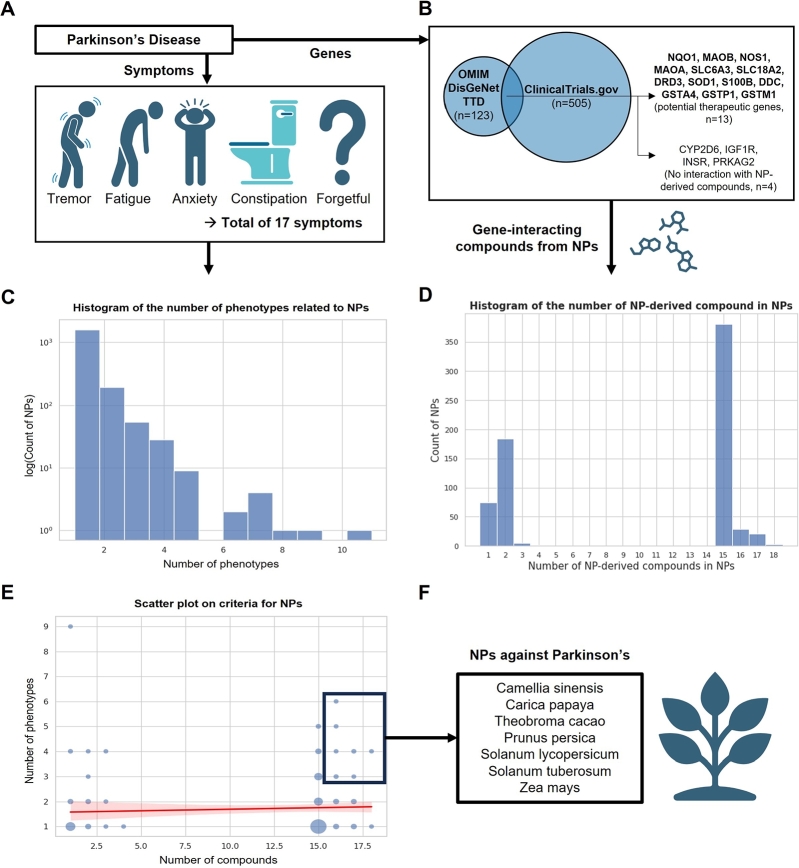
Identification of natural products (NPs) for treatment of Parkinson's disease (A-B) Identification of phenotypes(symptoms) and genes associated with the Parkinson's disease. (C) Histogram of NPs that are associated with disease-related phenotypes. The x-axis is the number of the associated phenotypes, and the y-axis is the count of NPs in log. (D) Histogram of NPs that contain compounds targeting disease-associated genes. The x-axis is the number of NP-derived compounds in NPs, and the y-axis is the count of NPs. (E) The correlation between the two approaches. The size of the blue circle is proportional to the number of NPs. The red line is the trend line. (F) The final top 10% NPs selected based on both criteria. (Created with the icons from BioRender.com)

In detail, a gene can (i) cause the heritable tendency to develop the Parkinson's disease (OMIM, DisGeNET), (ii) be a known therapeutic target (TTD, DisGeNET), (iii) or serve as a diagnostic or indicative biomarker (DisGeNET). From each database, the genes related to Parkinson's disease were searched, either by “Parkinson's disease” or by its UMLS CUI, ‘C0030567’. Then, the union gene set from all three databases was then acquired. Additionally, we obtained the list of genes which are used as therapeutic targets for Parkinson's disease in clinical trials through ClinicalTrials.gov [32]. The total of 17 genes present in both the disease-gene databases and the ClinicalTrials repository were selected as potential therapeutic target genes for further analyses (Fig. 2B).

### 2.3 Identification of compounds that influence the therapeutic genes

In order to find the compounds that can influence disease-related genes, the compound-gene relations in the COCONUT database [30] were used. Only the relations with experimental or clinical support were selected. The original sources for the relations were from DCDB [33], BindingDB [34], DrugBank [35], and in-house experimental data. From these relations, only the compounds that influence (i.e. target) the disease-related genes were acquired.

### 2.4 Identification of natural products that contain specific compounds

To find the natural products (NPs) that are known to contain the compounds that target the disease-related genes, the natural product-compound relations in the COCONUT database [30] were used. Only the relations with the experimental support were selected. The original source for the relations were from KTKP [36], TCMID [37], CMAUP [38], TCM-ID [39], FooDB [29], NPASS [40], and in-house experimental data. From these relations, only the NPs that contain the compounds that target the disease-related genes were acquired.

### 2.5 Selection of natural products

The selection of the NPs that can potentially treat Parkinson's disease were based on both the aforementioned disease-related phenotypes and the potential therapeutic target genes (see the sections above). The COCONUT database [30] were used for this part of the analysis, as it contains extensively detailed records on TOMs. Hence, the database could provide which NPs were used to treat certain symptoms according to TOM, as well as the detailed composition of compounds found in each NP. The selection process involved two independent criteria: the number of phenotypes that an NP targets, and that of compounds in an NP that influence therapeutic target genes.

From 2,288,893 NPs available in COCONUT [30], 1,957 of them were found to be used in the treatment for at least one of the disease-related phenotypes. The NPs were then ranked based on the number of the associated phenotypes, to find the NPs that deal with the most cases of the symptoms. Similarly, from 64,966,141 compounds available in COCONUT, 56,266 compounds were found in at least one NP. From these NP-derived compounds, 30 compounds were known to interact (i.e. target) with at least one of the potential therapeutic genes. Likewise, the NPs were again ranked based on the number of associated compounds contained in each NP, to find the NPs that can target the most number of the genes. Finally, we selected the NPs that are ranked within the top 10% on both the criteria (Fig. 2E-F). The very compounds found in these NPs were used for the further analysis.

### 2.6 Toxicity prediction analysis for drug candidates

The NPs and NP-derived compounds were considered for the next step of the pipeline, only if their toxicity levels were predicted to be within the range of the majority (95%) of the approved drugs.

The toxicity prediction was performed with Toxicity Estimation Software Tool (TEST), developed by the Environmental Protection Agency (EPA). The tool utilizes the quantitative structure activity relationships (QSARs) methodologies to create a model to that can estimate the toxicity based on the molecular descriptors (e.g. molecular weight) of given compounds.

Briefly, given a set of compounds-of-interest, their SMILES descriptors were acquired from PubChem repository to use as the inputs. With the exception of the compounds whose SMILES could not be recognized by the TEST, the following toxicity endpoints were calculated and recorded for each compound: “48-hour Daphnia magna (DM) 50% lethal concentration ($LC_{50}$)”, “96-hour Fathead minnow (FM) $LC_{50}$”, “Developmental Toxicity (DTox)”, and “Ames Mutagenicity (AMut)”. For the $LC_{50}$ endpoints, the lower values indicate higher toxicity, while for the DTox and AMut, the higher values indicate higher probability of toxicity.

To create a toxicity distribution curve for each of the endpoints, we applied TEST on the SMILES descriptors of the drugs against neurological diseases, from DrugBank [35]. Only the drugs whose market statuses are “Approved”, “Withdrawn”, or “Investigational” were selected, in order to reasonably consider therapeutically meaningful cases only. The neurological diseases were defined as the diseases that are classified as one of the following categories from DisGeNET: “Behavior and Behavior Mechanisms”, “Mental Disorders”, and “Nervous System Diseases”.

After running TEST on the drug candidate as well, its predicted toxicity endpoint values were converted into z-scores, based on the distribution of the approved neurology medications. The z-scores were than used to determine whether the candidate was not within the low 5% for the $LC_{50}$s, or the high 5% for the genetic toxicity.

### 2.7 Pharmacokinetic analysis for drug candidates

For the pharmacokinetics (PK) analysis, we utilized the “absorption, distribution, metabolism, excretion, and toxicity (ADMET)” scores predicted using admetSAR [41] available in DrugBank [35].

These values were used to indirectly assess whether a given NP-derived compound is expected to be delivered to intended targets. The ADMET metrics acquired were the following: human intestinal absorption value and probability, blood brain barrier value and probability, Caco-2 permeability value and probability.

## 3 Results

### 3.1 Case study: Parkinson's disease

To illustrate that the NaCTR pipeline can work, we chose Parkinson's disease, a progressive neurodegenerative disease [31], as an example subject for case study. The disease is characterized by its specific set of neuropathology, including neuroinflammation, mitochondrial dysfunction, and abnormal accumulation of *α*-synuclein (i.e. “Lewy body”), which is ultimately associated with the loss of dopaminergic neurons [31], [42]. This loss causes various symptoms of Parkinson's disease, including motor symptoms such as rest tremor, rigidity, and hypokinesia, as well as non-motor symptoms such as sleep disorders, emotional problems, and dementia.

The disease was selected for a number of its adequate qualities. First, according to prevalence studies, the Parkinson's disease is known to affect more than 1% of the population over 65 years of age [43], [44]. Hence, it is a serious illness that can generate extensive financial and socioeconomic burden to many [45], [46]. Secondly, the current treatment with the FDA-approved drugs focuses on alleviating the symptoms by elevating the dopamine levels only (e.g. by levodopa), not by tackling the root of the problem [31], [35]. This implies that the patients need to continuously take the medication for temporal symptom relief, all the while the long-term use of the drugs is associated with severe side-effects [47]. Thus, overall, there is an urgent need for drug discovery that can provide fundamental treatment for Parkinson's disease. In addition, even though Parkinson's disease was claimed to have been observed in the past [48], [49], [50], [51], [52], the TOM to actually treat the disease is not yet clearly defined. Parkinson's disease was therefore an ideal target disease to test the applicability for NaCTR.

### 3.2 Identification of natural products for Parkinson's disease treatment

An important aspect of NaCTR to effectively decrease the search space is the initial search for the most potentially therapeutic natural products (NPs). This is achieved by an accumulation of evidences in various aspects of TOM. The initial two TOM-based features utilized are: the Parkinson's disease-related phenotypes that NPs are known to treat, and the disease-related genes that the compounds contained in NPs are known to interact with.

#### 3.2.1 Finding natural products that can treat Parkinson's disease-related symptoms

For the first feature, the reduction of Parkinson's disease into its related phenotypes(symptoms) was achieved by a literature survey (Fig. 2A; see Materials and Methods). The rationales behind this approach were that, the expansion of the phenotype into the related symptoms allows effective translation of the disease into the terms that are available in TOM. This idea is further supported by a previous study that showed similar phenotypes were treated by the similar sets of drugs [53]. Hence, higher the number of the disease-related phenotypes that a given NP was known to treat, more likely would it be for the NP to treat the Parkinson's disease.

The total of 17 NP-treatable phenotypes were identified to be related to Parkinson's disease (Fig. 2A, supplementary table 1). According to the COCONUT database [30], 1,957 NPs were associated with these phenotypes. On average, NPs were associated with 1.3 phenotypes, with a range from 1 to 12 phenotypes per NP (Fig. 2C, supplementary table 2).

#### 3.2.2 Finding natural products that can influence Parkinson's disease-related genes

For the second feature dealing with the disease-related genes, we collected the genes associated with Parkinson's disease from various databases (See Materials and Methods). The databases were carefully selected to cover many aspects that a gene could be involved in the disease; namely, the genes collected held heritable, therapeutic, or diagnostic values for the disease. As these genes are either potential sources or consequences for the disease, externally influencing the genes can be effective for the treatment, and were aptly labeled as the ‘therapeutic genes’. Especially, only the genes that were involved in the past clinical trials were furthermore selected to make sure that targeting these genes could have even better therapeutic possibilities. There were in total 17 genes that satisfied this criteria: CYP2D6, DDC, DRD3, GSTA4, GSTM1, GSTP1, IGF1R, INSR, MAOA, MAOB, S100B, SLC6A3, SLC18A2, SOD1, PRKAG2, NOS1, and NQO1 (Fig. 2B).

Finding the compounds that are known to influence these genes can hold therapeutic effects against the disease. Such influence could be by binding directly onto the expressed proteins or by affecting the expression levels of the genes. Therefore, the compound-gene relations that are available in the COCONUT [30] were utilized to parse out the compounds that can influence (i.e. target) these genes (See Materials and Methods). There were 30 compounds that satisfy this criteria (supplementary table 3).

The NPs that contain these compounds can therefore target the potential therapeutic genes. Through natural product-compound relations in the COCONUT [30], we were able to find 698 NPs that contain at least one of such compounds (supplementary table 3). In this process, four genes (CYP2D6, IGF1R, INSR, PRKAG2) were excluded from the target genes list, since they were not targets of any NP-derived compounds, thereby leaving 13 genes. Out of the 698 NPs, 433 NPs contained sets of compounds that can each target all 13 genes. Each NP had between 1 and 18 compounds each, with an average of 10.1 compounds (Fig. 2D, supplementary table 4).

#### 3.2.3 Finding natural products that effectively target both symptoms and genes

The NPs, which are known to treat Parkinson's disease-related symptoms, and which simultaneously contain compounds that can target the disease-related genes, were acquired by finding the intersection from the both criteria, resulting in 288 NPs.

There was a weak (0.17), but significant (p-value of 0.0088) Spearman's correlation between the numbers of supporting evidences from both criteria (Fig. 2E). Such correlation between the independent approaches suggested that higher number of therapeutic compounds could prove effective against higher number of phenotypes. Hence, the NPs that rank high in both aspects can be considered as therapeutic NP candidates.

By narrowing down to the top 10% of NPs from both approaches, we identified seven NPs (Camellia sinensis, Carica papaya, Theobroma cacao, Prunus persica, Solanum lycopersicum, Solanum tuberosum, Zea mays). Most of the NPs (except Solanum tuberosum and Zea mays) were found to be associated with Parkinson's disease or to have neuroprotective roles [54], [55], [56], [57], [58], [59] (Fig. 2E-F). There were 19 compounds available from those seven NPs (supplementary table 6). Among these, ions (calcium and zinc) and compounds without identifiable SMILES information (tumeric oil) were eliminated, leaving 16 compounds for the subsequent analyses.

### 3.3 Toxicity analysis for NP-derived compounds

The toxicity level for 16 NP-derived compounds in the seven NPs was estimated by running its SMILES descriptor in the TEST, a publicly available tool from EPA. The tool was developed for environmental toxicity test, but was selected for the following reasons.

The tool's predicted values ($LC_{50}$ values in four model organisms, developmental and mutagenic toxicity values; see Materials and Methods) indicated toxicity that can be generalized among all drugs. The usual toxicity testing for drugs are done using a variety of criteria that each require different experimental procedures and threshold [60], which are mostly not available for NP-derived compounds. The TEST tool allowed the prediction of toxicity simply by using SMILES. Hence, we could create reference values of safety using all available drugs for neurological diseases, and use those to infer the potential toxicity for our compounds of interest. It is worth noting that there were indeed some website-based tools that allow toxicity values that are more focused on specific side-effects upon therapeutic ingestion [61], [62], [63], but these tools were either not capable or efficient in bulky prediction of the toxicity. The TEST tool excelled in bulky analyses, allowing for more reliable prediction among hundreds of drugs. Finally, all commercial drugs are toxic in certain degree, and it is only when they are taken at the correct level do they exhibit the intended therapeutic effects [64]. Even one of the most well-known and widely used drugs like ibuprofen or Tylenol have side effects [65], [66]. Thus, it would not make sense to simply reject potentially therapeutic compounds by mere side effect predictions. We therefore decided to only get rid of compounds, if they are found to be toxic at the extreme level (e.g. top 5%) compared to all other currently available drugs.

**Fig. 3:**
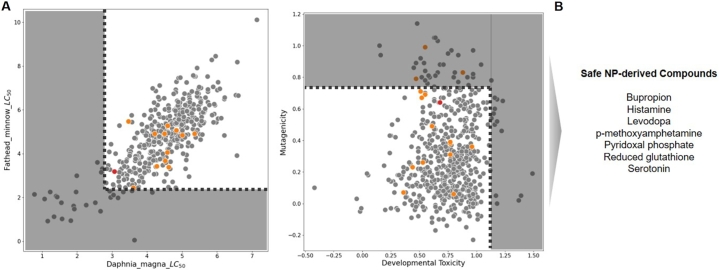
Scatter plots showing the distribution of the predicted values of toxicity for paired endpoints available in TEST. The dashed lines indicate the top 5% of high toxicity, and the shaded area indicate the rejected values. The color indicates compound types: grey - drugs for neurological disorders, orange - NP-derived compounds, red - glutathione (A) “Daphnia magna *LC*_50_” and “Fathead minnow *LC*_50_” and “Developmental Toxicity” and “Mutagenicity”. (B) A list of 7 NP-derived compounds that passed all the toxicity criteria.

Hence, the TEST-based toxicity values for the 16 NP-derived compounds were first calculated (supplementary table 7). Then, to observe these values under a meaningful reference, we ran the same set of analysis on a list of drugs for neurological diseases in DrugBank, and acquired the distribution of the predicted values (Fig. 3). Out of the 708 medications, the SMILES structures of 600 molecules could be recognized by the TEST. Based on the z-score, the bottom 5% (z=-1.645) for the $LC_{50}$ values were 2.77 and 2.40 for DM and FM, respectively, while the top 5% (z=1.645) for the genetic toxicity were at 1.11 and 0.73 for DTox and AMut, respectively (Fig. 3A).

We could see, from the distribution of the all neurological medications, that seven out of the 16 NP-derived compounds were well-within the 95% range in terms of various contexts of toxicity (Fig. 3B and supplementary table 7). Hence, these compounds were considered as relatively safe candidates to investigate further into.

### 3.4 Pharmacokinetic criteria on NP-derived compounds

The final step in the NaCTR NP-derived drug discovery pipeline involved the pharmacokinetic (PK) characterization. PK approaches to drug analyses often require various experimental investigation with different criteria. As in toxicity analyses, the experimental results for the PK properties were not readily available, especially for the NP-derived compounds we found. We therefore decided to rely on the predicted values by admetSAR [41] to estimate the PK properties.

**Fig. 4:**
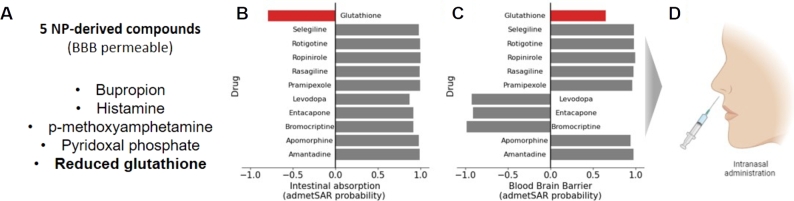
The pharmacokinetic analysis of NP-derived compounds. (A) The 5 NP-derived compounds that passed the toxicity and the pharmacokinetics test. The predicted admetSAR probability values of (B) human intestinal absorption and (C) blood-brain barrier of glutathione and approved drugs for Parkinson's disease. (D) The recommended administration method (intranasal) inferred from the predicted admetSAR was depicted. (Created with the icons from BioRender.com)

To first check if the values present in the ADMET model were consistent with the actual administration profiles, we assessed the intestinal absorption and blood-brain barrier (BBB) probability values predicted for 10 FDA-approved Parkinson's disease drugs (Fig. 4B-C). Most FDA-approved drugs for Parkinson's disease were administered orally and were permeable to the BBB, as indicated by the predicted admetSAR values. The exceptions to the BBB included levodopa, bromocriptine, and entacapone, which were known to require specific channels to cross the barrier or act peripherally [67], [68], [69]. As the predictions aligned with the drugs' profiles, we could safely assume that PK predictions were reliable. Out of the seven compounds that passed the toxicity criteria, five compounds were predicted to cross the BBB (Fig. 4A), as the final NP-derived drug candidates to treat the Parkinson's disease.

The relations between the seven NPs and the qualified compounds, between the NPs and disease-related phenotypes, and between the compounds and the potential target genes are summarized in supplementary tables 9, 10, and 11.

### 3.5 Literature evidences for NP-derived compounds

In addition to the fact that the suggested NPs were indeed known for their neuroprotective properties [54], [55], [56], [57], [58], [59], we found that these NPs shared most of the qualified compounds. Furthermore, the enrichment test on the compounds' potential therapeutic target genes revealed involvement in the oxidative stress-related processes, such as biological oxidations and glutathione conjugation [70]. This also aligned with findings from previous NP studies [54], [57].

For a more detailed analysis, we conducted an in-depth analysis on one example compound, reduced glutathione, which was commonly found in the seven NPs and was most closely related to oxidative stress. First, the prediction result of reduced glutathione indicates that it is permeable to the blood-brain barrier but has low oral bioavailability (Red bars in Fig. 4B, 4C). These findings are consistent with the known property of glutathione. Glutathione is, therefore, typically administered intravenously or intranasally [71].

**Fig. 5:**
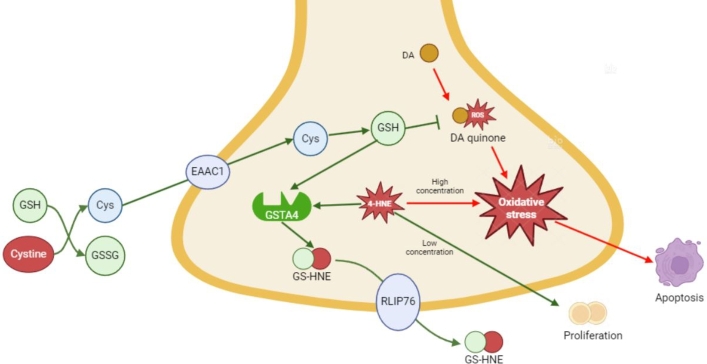
Suggested MOA model for GSH. The figure is the schematic illustration of the neuroprotective mechanism of glutathione for Parkinson's disease. abbreviation: GSH, glutathione; GSSG, oxidized glutathione; Cys, cysteine; DA, dopamine; ROS, reactive oxygen species. (Created in BioRender.com)

Next, we surveyed literature evidence to hypothesize the mechanism of action (MOA) of glutathione for Parkinson's disease (Fig. 5). Glutathione, a tripeptide consisting of glutamate, cysteine, and glycine, is an antioxidant that protects cells from oxidative stress. The injection of glutathione elevates cysteine levels outside cells by reducing cystine to cysteine. The cysteine is then transported into cells and synthesized into glutathione [72]. The synthesized glutathione is known to protect cells from oxidative stress through various mechanisms. Firstly, it binds with dopamine quinone, an auto-oxidized form of dopamine, to reduce oxidative stress [72], [73]. Dopamine quinone is a mediator of oxidative stress, by binding to alpha-synucleins to stabilize the alpha-synuclein protofibrils [74]. Another mechanism involves preventing oxidative stress through GSTA4, a potential therapeutic target we suggested [75]. 4-Hydroxynonenal (4-HNE) is an end product of lipid peroxidation and a consequence of aerobic metabolism. High levels of 4-HNE induce oxidative stress signaling pathways, such as apoptosis. GSTA4 conjugates 4-HNE to glutathione for detoxification. The glutathione conjugate of 4-HNE (GS-HNE) is transported out of the cell through ATP-dependent transport by RLIP76. Interestingly, low levels of 4-HNE, modulated by glutathione and GSTA4, induce proliferation. These protective mechanisms indicate the potential of glutathione as a novel drug for Parkinson's disease.

Finally, there are clinical trials of glutathione for Parkinson's disease that support the suggested MOA [71], [76], [77], [78]. As we predicted low oral bioavailability of glutathione, the clinical trials administered it intravenously [76] and intranasally [71], [77], [78]. Both types of administration showed promising effects in early clinical trials. However, some intranasal administration trials showed that while glutathione improved Parkinson's disease symptoms, it was not superior to the placebo effect after a three-month intervention [77]. These clinical trial results indicate the potential of glutathione as a novel therapeutic agent for Parkinson's disease but also highlight the need for further investigation and additional clinical trials to confirm its efficacy.

## 4 Discussion

The drug discovery pipelines require enormous time and cost with a risk of failure. Hence, various approaches are adopted in drug discovery to reduce the cost and risk. One approach is an NP-derived drug. The advantages of NPs have been verified through human history [9], [10]. Reflecting this, more than half of the small molecule drugs developed since 1981 have been derived from NPs [17]. Another approach is *in silico* drug discovery. Recently, *in silico* drug discovery has come to the fore, reducing the time and cost associated with drug development. Therefore, we developed a NaCTR, a *in silico* NP-derived drug discovery pipeline.

We have chosen Parkinson's disease, which is the second most common neurodegenerative disease in the world, as a case study for the NaCTR pipeline. Despite its high prevalence, there are no fundamental treatments for Parkinson's disease. Hence, the discovery of novel therapeutic agents for Parkinson's disease is a crucial challenge. Nevertheless, Parkinson's disease lacked extensive experimental data, and faces obstacles in adapting existing *in silico* models. Therefore we chose Parkinson's disease, which is suitable to utilize the NaCTR pipeline, a database-based data-driven approach.

With the NaCTR pipeline, we successfully illustrated how the utilization of both traditional oriental medicine (TOM) to effectively search for therapeutic compounds from NPs, through reduction of search space. The potential therapeutic NPs were acquired through two approaches, identifying NPs that are associated with disease-related phenotypes and those that contain compounds targeting therapeutic target genes. For the former approach, we identified NPs that have connections with disease-related phenotypes from the NP database. For the latter approach, we identified potential therapeutic targets from disease-related databases and omics databases. Then, we searched NPs that contained compounds related to these potential therapeutic targets from drug-related databases. We selected NPs that ranked highly in both approaches. We then utilized a toxicity prediction tool to assess the safety of NP-derived compounds. Finally, we analyzed the pharmacokinetic profiles of the NP-derived compound to assess the efficacy of the NPs. As a result, we proposed five NP-derived compounds from seven NPs as a treatment for Parkinson's disease.

**Figure S1:**
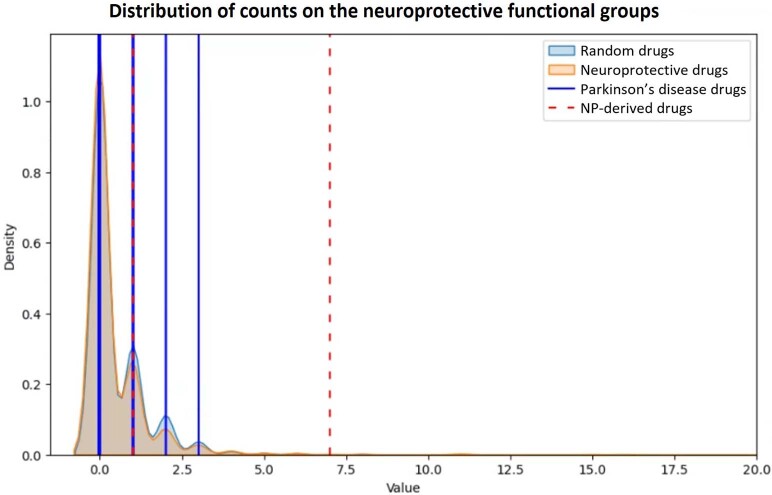
Distribution graph of neuroprotective functional groups on drugs. The values for the qualified NP-derived compounds are shown in red dashed line. The values for the known Parkinson's disease drugs are shown in blue solid lines. The distribution of the counts for random drugs and neuroprotective drugs are shown as shaded areas in blue and orange, respectively.

The mechanism of action from the literature survey suggests the antioxidant function of the NPs and NP-derived compounds in cellular environments. Our findings align with previous studies [54], [55], [56], [57], [58], [59], [72], [73], indicating the reliability of our pipeline. It is worth mentioning that the five NP-derived compounds, which were determined as the potential therapeutic compounds for the treatment of Parkinson's disease, all had at least one of the functional groups known to have neuroprotective properties: phenolic [79], carbonyl [80], carboxylic [81], [82], and amine [83] groups (supplementary table 12). The number of functional groups for these NP-derived compounds were significantly higher (p-value<0.01) compared to those of the random drugs available in DrugBank (supplementary figure 1). The count was especially high (i.e. detection of 7 functional groups) for the example case study compound, the reduced glutathione. This is further noteworthy, since the current main treatment for the disease, levodopa, also had the high number (5) of the neuroprotective functional groups. As the NaCTR pipeline only concerns the potential toxicity with chemical structural information, this additional therapeutic support even further highlights the validity of the pipeline.

There are several limitations to our pipeline. The first limitation lies in identifying potential therapeutic target genes. NaCTR is based on the database-driven approach. The genetic basis of Parkinson's disease, which we used as a case study, is not yet fully understood. As a result, the information available in each database is limited. To overcome this, we utilized multiple pieces of evidence from various databases. This is an area that is expected to improve as our understanding of Parkinson's disease advances. In addition, extensive drug perturbation data like Connectivity Map [84] does not exist for Parkinson's disease. The lack of such data hindered the adoption of existing pharmacokinetic/pharmacodynamic (PK/PD) models with dosage levels. Thus, we conducted PK analyses based on the available predicted values. We expect that these limitations, especially on PD, can be addressed as more experimental data on Parkinson's disease accumulates in the future.

Despite the above limitations, we proposed seven NPs (Camellia sinensis, Carica papaya, Theobroma cacao, Prunus persica, Solanum lycopersicum, Solanum tuberosum, Zea mays), and a promising NP-derived drug candidate, glutathione, along with four others. The methods applied in our pipeline are relatively simple but effective in finding an NP-derived novel drug candidate. Furthermore, the NaCTR pipeline also proposed the mechanism of action of NPs and NP-derived compounds for Parkinson's disease, providing necessary information for further clinical trials. Finally, the simplicity of our pipeline enabled drug discovery for Parkinson's disease, which lacked extensive drug perturbation data. We expect to adopt our pipeline for other diseases that also lack extensive drug perturbation data.

## Funding

This work was supported by the 10.13039/501100014188Ministry of Science and ICT through the National Research Foundation (RS-2023-00262747). The funders had no role in study design, data collection and analysis, decision to publish, or preparation of the manuscript.

## CRediT authorship contribution statement

**Seunghwan Jung:** Writing – original draft, Visualization, Validation, Software, Resources, Project administration, Methodology, Investigation, Funding acquisition, Formal analysis, Data curation, Conceptualization. **Kwansoo Kim:** Writing – review & editing, Writing – original draft, Visualization, Validation, Supervision, Software, Resources, Methodology, Investigation, Formal analysis, Data curation, Conceptualization. **Seunghyun Wang:** Writing – original draft, Visualization, Software, Resources, Methodology, Investigation, Data curation, Conceptualization. **Manyoung Han:** Writing – review & editing, Writing – original draft, Visualization, Investigation. **Doheon Lee:** Writing – review & editing, Writing – original draft, Supervision, Project administration, Funding acquisition, Conceptualization.

## Declaration of Competing Interest

The authors declare that they have no conflict of interest.

## Data Availability

The codes used for analyses in the current study are available in the GitHub repository (https://github.com/sktoyo/NaCTR). The datasets used and/or analyzed are available from the corresponding author on reasonable request.

## Appendix A Supplementary material

MMC 1 Natural products (NPs) and Parkinson's disease phenotypes that NPs are known to treat. MMC 1

MMC 2 NPs and the count of Parkinson's disease phenotypes that NPs are known to treat. MMC 2

MMC 3 NPs, compounds they contain, and Parkinson's disease therapeutic genes that the compounds are known to target. MMC 3

MMC 4 NPs and the count of compounds contained that are known to target Parkinson's disease therapeutic genes. MMC 4

MMC 5 NPs and the counts of both the related phenotypes and the target genes. MMC 5

MMC 6 Top 10% NPs and their compounds based on the counts of both the related phenotypes and the target genes. MMC 6

MMC 7 Top 10% NP-derived compounds and their toxicity. MMC 7

MMC 8 Top 10% NP-derived compounds and their ADME values from DrugBank. MMC 8

MMC 9 The selected NPs and Parkinson's disease phenotypes that NPs are known to treat. MMC 9

MMC 10 The selected NPs and compounds contained that are known to target Parkinson's disease therapeutic genes. MMC 10

MMC 11 Qualified compounds and the Parkinson's disease therapeutic genes that they are known to target. MMC 11

MMC 12 Neuroprotective functional groups analysis on the qualified compounds. MMC 12
